# Preservation of Anatomical Preparations by Arsenuretted Hydrogen Gas

**Published:** 1851-05

**Authors:** 


### ANATOMY AND PHYSIOLOGY.

*Preservation of Anatomical Preparations by Arsenuretted Hydrogen Gas.*—Mr. MIDDLETON stated that, from the great expense of spirit of wine, and the various objections to the employment of other preservative fluids, he had been led to make some experiments with gases having antiseptic powers. He had found arsenuretted hydrogen to answer admirably, and he exhibited a specimen of diseased structure so preserved. The preparation had been several months in the bottle without showing the slightest symptom of change.

The object to be preserved should be placed in a suitable bottle filled with water. The bottle is then to be inverted in a pneumatic trough, and the gas passed into it in the ordinary way, so as completely to displace the water. The mouth of the bottle must then be closed with a good cork under water, and afterwards dipped in melted wax.

The gas can very well be prepared by mixing in a retort sulphuric acid, with a certain quantity of water.

Dr. King suggested that, for greater safety, the mouth of the bottle should be closed with a glass stopper, and hermetically sealed by the blow-pipe.—*London Medical Gazette.*

